# Multiple Respiratory Virus Detection in Acute Respiratory Infection Patients in Mie Prefecture, Japan, 2021–2023

**DOI:** 10.3390/v17030331

**Published:** 2025-02-27

**Authors:** Yuriko Tomita, Hiyori Okura, Rika Mochizuki, Manami Negoro, Takuya Yano, Yusuke Kobayashi, Ikuyo Takayama, Kiyosu Taniguchi, Shinji Watanabe, Hideki Hasegawa

**Affiliations:** 1 Research Center for Influenza and Respiratory Viruses, National Institute of Infectious Diseases, Tokyo 208-0011, Japani-taka@niid.go.jp (I.T.); hasegawa@niid.go.jp (H.H.); 2 Institute for Clinical Research, National Hospital Organization, Mie National Hospital, Tsu-shi 514-0125, Japan; negoro.manami.zt@mail.hosp.go.jp; 3 Mie Prefecture Health and Environment Research Institute, Yokkaichi 512-1211, Japan; 4 Center for Surveillance, Immunization and Epidemiologic Research, National Institute of Infectious Diseases, Tokyo 162-8640, Japan; 5 Department of Pediatrics, National Hospital Organization Mie National Hospital, Tsu-shi 514-0125, Japan; tngk7g04@gmail.com

**Keywords:** ARI surveillance, COVID-19, multiple virus infection

## Abstract

The Coronavirus disease 2019 (COVID-19) pandemic significantly impacted the circulation patterns of respiratory viruses worldwide. To better understand viral circulation patterns during the transition from pandemic to endemic phase, we conducted comprehensive respiratory virus surveillance in Mie Prefecture, Japan, during 2021–2023, coinciding with the Delta-to-Omicron transition of Severe Acute Respiratory Syndrome Coronavirus 2 (SARS-CoV-2). We collected respiratory specimens from acute respiratory infection patients in medical institutions, detecting 19 respiratory viruses using real-time PCR in 1573 valid samples out of 1605 specimens. Demographic and clinical data were available for some specimens. SARS-CoV-2 Omicron strains showed a peak positivity of 15–25% during the epidemic, while respiratory syncytial virus and human rhinovirus/enterovirus exhibited one to two annual epidemic peaks up to 57%, and human adenovirus maintained a positivity rate of 5–20% throughout the year. Age-dependent analysis revealed the significant detection of multiple viruses, particularly in children under 2 years, with up to six viruses detected simultaneously in those under 5 years. Our findings demonstrate varied respiratory virus prevalence patterns, with some viruses remaining active during the Omicron epidemic, suggesting its limited impact on other viruses. This comprehensive approach should enhance the understanding of respiratory virus epidemic dynamics and inform public health strategies.

## 1. Introduction

Respiratory infections are significant public health concerns, affecting individuals across all age groups, from infants to the elderly [1]. These infections are caused by various pathogens, including bacteria and viruses. Among viral pathogens are influenza viruses, respiratory syncytial virus (RSV), human metapneumovirus type A/B (HMPV), human rhinovirus (HRV), human parainfluenza virus type 1–4 (HPIV1–4), human coronaviruses (HCoV-NL63, HCoV-HKU1, HCoV-229E, HCoV-OC43), human bocavirus (HBoV), human adenovirus (HAdV), enterovirus (EV) and human parechovirus (HPeV) [2]. In vulnerable populations, such as infants, older adults and individuals with underlying health conditions, these infections can lead to severe illness, necessitating focused attention and care [3,4].

The Coronavirus disease 2019 (COVID-19) pandemic has significantly impacted the epidemiology of respiratory viruses worldwide. Global surveillance studies have documented unprecedented changes in respiratory virus circulation patterns during this period. Multiple studies across different regions demonstrated dramatic reductions in the detection rates of common respiratory viruses during periods of strict non-pharmaceutical interventions (NPIs) [5,6]. Surveillance data from various countries showed markedly reduced detection rates of common respiratory viruses including influenza viruses, RSV, HMPV and seasonal coronaviruses during the initial pandemic phase [7,8]. Following the relaxation of restrictions, distinct patterns emerged for different viruses. While influenza remained suppressed in many regions, RSV showed notable off-season resurgences with altered transmission dynamics [9,10]. These changes varied geographically, with some areas experiencing more severe outbreaks than typical seasonal epidemics, particularly affecting different age groups than usually observed [11,12]. The unprecedented global implementation of NPIs demonstrated that effective interventions are possible, while also revealing the inherent characteristics of respiratory viruses—their high transmissibility and capacity to evolve and cause epidemics rapidly [13]. These findings emphasize that a comprehensive understanding of the epidemic dynamics of each respiratory virus is crucial for effective management and prevention. Such an understanding is particularly essential for viruses with available or developing vaccines, as it enables the planning and implementation of targeted vaccination strategies [9].

Robust surveillance systems are therefore essential for monitoring these epidemiological changing patterns. Such systems should be capable of detecting regional variations, age-specific transmission trends and emerging variants in real-time. This information is vital for the efficient allocation of healthcare resources, optimization of public health policies and implementation of timely interventions [14].

In Japan, a surveillance system exists for reporting positive cases of influenza virus and RSV infections at sentinel sites [15]. However, this system lacks the capability to determine pathogen positivity rate and comprehensively analyze epidemic trends [16]. This limitation is particularly significant during periods with the concurrent circulation of Severe Acute Respiratory Syndrome Coronavirus 2 (SARS-CoV-2) and other respiratory viruses. A more comprehensive surveillance system capable of detecting multiple respiratory viruses and calculating positivity rates could offer valuable insights into the seasonal patterns, regional variations and age-specific infection dynamics [17].

In this study, we conducted comprehensive surveillance of respiratory viruses in Mie Prefecture, Japan, using specimens collected from outpatients with acute respiratory infection (ARI) between 2021 and 2023. We detected multiple respiratory viruses and calculated the positivity rate to elucidate detailed epidemic patterns. Clinical data from patients were also collected, and the frequency of respiratory symptoms was analyzed.

The results of this study are expected to provide valuable insights into the epidemic dynamics of respiratory viruses in Mie Prefecture and elucidate the epidemiological patterns of concurrent respiratory viruses during the transition of SARS-CoV-2 from pandemic to endemic status.

## 2. Materials and Methods

### 2.1. Ethics Statement

This study adhered to the Declaration of Helsinki and was approved by the Ethics Committee of the National Institute of Infectious Disease (approval number: 1291, approval date: 19 November 2021). We used anonymized specimens obtained from outpatient clinics of collaborating hospitals. These specimens were collected during routine medical care. Oral consent was obtained from patients or their parents for sending specimens to the National Institute of Infectious Diseases. Information about the use of residual specimens for respiratory virus research was provided through hospital websites and in-hospital notices, with an opt-out option available.

### 2.2. Collection of Specimens

**Figure 1:**
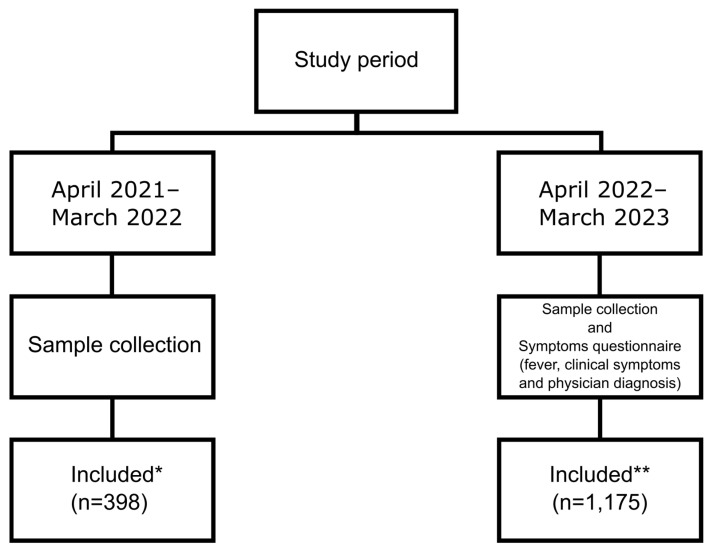
Period of specimen collection, information collected and number of specimens included in this study. Twenty samples collected during April 2021 and March 2022 (*) and twelve samples collected during April 2022 and March 2023 (**) were excluded from this analysis (see Results for details).

**Figure 2:**
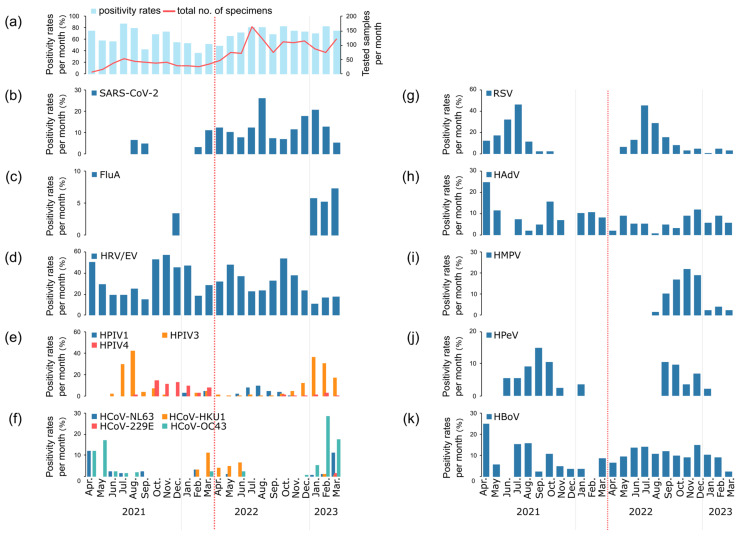
Monthly distribution of respiratory viruses in Mie Prefecture from April 2021 to March 2023. (**a**) Total specimens tested (red lines, right *y*-axis) and overall positivity rates (right blue bar, left *y*-axis). (**b**–**k**) Positivity rates for individual viruses: (**b**) SARS-CoV-2; (**c**) influenza virus type A (FluA); (**d**) human rhinovirus/enterovirus (HRV/EV); (**e**) human parainfluenza virus types 1, 3 and 4 (HPIV1, 3, 4); (**f**) human coronaviruses (HCoV-NL63, HCoV-HKU1, HCoV-229E, HCoV-OC43); (**g**) respiratory syncytial virus (RSV); (**h**) human adenovirus (HAdV); (**i**) human metapneumovirus A/B (HMPV); (**j**) human parechovirus (HPeV) and (**k**) human bocavirus (HBoV). Influenza virus A H1N1pdm09 (FluA/H1N1pdm), influenza type B (FluB) and human parainfluenza type 2 were not detected. Positivity rates are presented as percentages on the left *y*-axis for all panels. The vertical red dashed lines indicate the change in the specimen collection method (see Figure 1 for details).

Respiratory specimens were collected from outpatients presenting with ARI symptoms who visited any of three hospitals or one private clinic in Mie Prefecture between April 2021 and March 2023. ARI was defined based on the following criteria: (1) the presence of at least one of four respiratory symptoms (cough, runny nose, sore throat, wheezing) and (2) clinical assessment by a physician indicating suspected ARI. Specimens (nasopharyngeal swabs or nasal discharge) were collected using a swab (#260999, Fujirebio Inc., Tokyo, Japan), suspended in 3 mL of transport medium (UT-300, Sugiyama-Gen Co., Ltd., Tokyo, Japan) and stored at −80 °C until nucleic acid extraction. The study period was divided into two phases (see Figure 1 and Figure 2). During the first phase (April 2021 to March 2022), respiratory specimens were collected from patients displaying ARI symptoms. In the second phase (April 2022 to March 2023), respiratory specimens were collected as in the first phase, but additional data were gathered using a symptom questionnaire. The collected data encompassed fever records (maximum body temperature), respiratory symptoms (presence or absence of cough, runny nose, sore throat, wheezing and general fatigue) and physician diagnosis (Figure 1).

### 2.3. Nucleic Acid Extraction and Real-Time PCR for Respiratory Virus Detection

Nucleic acid extraction was performed using the MagMAX Viral/Pathogen Nucleic Acid Isolation Kit (Cat # A48310; Thermo Fisher Scientific, Waltham, MA, USA) with 200 µL of specimen and a 60 µL elution volume. The FTD Respiratory Pathogens 21 Plus Kit (Cat #: 11373924; Siemens, Munich, Germany) was used to detect multiple respiratory viruses using pathogen-specific primer/probe sets: influenza viruses [influenza type A (FluA), influenza virus A H1N1pdm09 (FluA/H1N1pdm), influenza type B (FluB)], HMPV, HRV, RSV, HPIV1–4, HCoV-229E, HCoV-NL63, HCoV-OC43, HCoV-HKU1, HBoV, HAdV, EV and HPeV. Analyses were performed according to the kit manual with a modified nucleic acid template volume of 5 µL. The efficiency of nucleic acid extraction was monitored using the kit’s internal control. Results were considered valid when negative controls remained below cycle threshold (Ct) and both positive and internal controls showed Ct values below 34. A sample was considered positive when the Ct value was below 40. When a specimen tested positive for either HRV or EV or both, it was considered as one detection. SARS-CoV-2 detection was performed using a LightCycler 480 (Roche Diagnostics, Basel, Switzerland) with one-step real-time PCR targeting the N gene using the N2 primer/probe sets, following the protocol described in Shirato et al. [18].

### 2.4. Statistical Analysis

The statistical software IBM SPSS Statistics (version 29.0.2.0, IBM Corporation, Armonk, NY, USA) was used for data analysis. Variables were analyzed in terms of number, percentage, medians, interquartile ranges (IQRs) and chi-square tests (significance, *p* < 0.05). Statistical significance for single and co-detections of each virus was assessed using odds ratios and 95% confidence intervals (CIs).

## 3. Results

### 3.1. Characterization of the Study Population and Specimen Collection

From April 2021 to March 2023, 1605 specimens were collected from patients presenting with ARI symptoms and real-time PCR was performed. Of these, 1573 specimens (98%) were considered valid and included in subsequent tabulations and analyses (Figure 1, “Included”). The remaining 32 specimens (2%) were excluded based on one of the following reasons: unable to test because of specimen problems, difficulties in result interpretation (such as undetectable real-time PCR internal controls) or unable to test because of a lack of extracted nucleic acid. The majority of specimens (57.2%) were from male patients, with children under 5 years comprising approximately 70%. The median age was 2.4 years [IQR: 1.1–5.8]. Of the 923 cases with a recorded body temperature, 83.9% exhibited fever ≥38 °C (Table 1).

The most prevalent clinical symptoms were cough (33.7%) and runny nose (24.2%), with bronchitis/bronchiolitis (20.5%) and upper respiratory inflammation (10.4%) being the most common physician diagnoses (Table 1).

### 3.2. Monthly Detection Numbers and Positivity Rates of Viruses

The number of specimens remained low between April 2021 and March 2022 due to the use of stored samples. Following the implementation of systematic sampling methods and the initiation of new specimen collection in April 2022, the number of specimens increased (Figure 2a). Overall, the positivity rate of detecting at least one virus ranged from 37.0 to 86.8% throughout the study period.

The number of SARS-CoV-2 detections between April 2021 and March 2022 remained low, at 1–5 cases per month, with a positivity rate of ~10% or less. This low detection rate can be attributed to the specimen collection protocols during this period, where specimens from SARS-CoV-2 antigen-positive cases were excluded from the study cohort. Consequently, the observed low positivity rate in this period likely reflects this systematic exclusion of antigen-positive cases rather than the true prevalence of SARS-CoV-2 infection. From 2022 onwards, the prevalence of the Delta strain declined, but the Omicron strain emerged and was most frequently detected in August 2022. HRV/EV and RSV demonstrated higher positivity rates (2–57% and 1–46%, respectively) compared to other respiratory viruses, while HAdV and HBoV were detected throughout the year. Influenza and HMPV were rarely detected until late 2022, when their detection rates increased. Notably, respiratory viruses other than influenza and HMPV remained prevalent during the SARS-CoV-2 Omicron epidemic.

### 3.3. Number, Percentage and Median Patient Age of Positive Samples by Age Group

The relationship between detected pathogens and patient age was also analyzed. Patients were divided into four age groups: <2, 2 to <5, 5 to <15 and ≥15 years. In 2021, specimens from SARS-CoV-2 antigen-positive cases were excluded from the study cohort due to the testing protocols. Therefore, specimens collected between April 2022 and March 2023 were the focus of this analysis (Table 2).

Age-dependent differences in respiratory virus detection patterns were observed. In the age groups <2 years and 2 to <5 years, HRV/EV and RSV showed high detection rates. Specifically, in infants <2 years, HRV/EV was detected in 32.2% and RSV in 17.5% of cases, whereas in the group aged 2 to <5 years, HRV/EV was found in 37.4% and RSV in 19.0% of cases. The detection rate of these viruses decreased in the age groups above 5 years, with SARS-CoV-2 exhibiting higher detection rates in the groups aged 5 to <15 years and ≥15 years. SARS-CoV-2 exhibited a unique detection pattern, showing a relatively low detection rate of 8.8% in the group aged 2 to <5 years, but comparatively high detection rates of 13.6–18.1% in the other age groups (Table 2).

The median age of detection was relatively high for SARS-CoV-2 and FluA (SARS-CoV-2, 2.7 years [IQR 0.8–8.9]; FluA, 5.8 years [IQR 3.3–9.8]), whereas the median age of detection for most other viruses was around 2 years. For example, the median age for HRV/EV was 2.2 years [IQR: 1.1–3.8 years] and for RSV it was 1.9 years [IQR: 1.0–2.8 years]. Furthermore, a notable characteristic of age-dependent detection was that over 60% of positive samples for each virus, except FluA and HCoV-229E, were obtained from children under 5 years of age. In particular, HBoV and HPeV were detected exclusively in children under 5 years of age, with only two exceptions. Detection rates for many viruses decreased in patients of age groups above 5 years (Table 2).

### 3.4. Number and Percentage of Co-Detected Viruses by Age Group

In infants and young children, the simultaneous detection of multiple respiratory viruses has been reported [19,20]. To further investigate this phenomenon, the co-detection of respiratory viruses by age group was examined (Table 3).

Analysis of virus co-detection rates in each age group revealed a significant difference among groups. For infants aged <2 years and 2 to <5 years, virus-negative samples were observed in 19.3% and 12.9%, respectively. By contrast, the groups aged 5 to <15 years and ≥15 years exhibited significantly higher rates of virus-negative samples, at 41.8% and 55.1%, respectively. Single-virus detection was predominant in all age groups except those aged ≥15 years. In children under 5 years of age, there were three cases in which five or six viruses were detected simultaneously. The percentage of cases with two or more viruses detected was approximately 25% in the <2 age group, compared with only 2.8% in the ≥15 age group. Furthermore, a notable difference was observed in the frequency of multiple virus detections across age groups. In specimens from patients aged <5 years, 21 specimens (2.49%) were detected with ≥4 viruses. Conversely, no instances of ≥4 viral detections were observed in specimens from patients aged ≥5 years (Table 3).

### 3.5. Proportions and Odds Ratios of Single Infections and Co-Infections for Each Respiratory Virus

During the early stages of the COVID-19 pandemic, a significant decrease in the detection rates of other respiratory viruses was reported concomitant with the global spread of SARS-CoV-2 [21]. To elucidate the infection patterns of respiratory viruses under these unprecedented circumstances, we compared the proportions of single detections and co-detections for each virus. Specifically, we aimed to test the hypothesis that SARS-CoV-2, the causative pathogen of COVID-19, exhibits a higher proportion of single infections compared with other respiratory viruses (Table 4).

The detection ratios, odds ratios and 95% CIs were calculated using the detection numbers of single detections and co-detections for each virus (Table 4). SARS-CoV-2 and FluA demonstrated strong tendencies toward single detection, with odds ratios of 0.18 (95% CI: 0.13–0.26) and 0.10 (95% CI: 0.03–0.30), respectively. By contrast, HBoV exhibited a significantly higher tendency for co-detection with other viruses, showing an odds ratio of 1.98 (95% CI: 1.20–3.26). No statistically significant differences were observed for HPIV4, HCoV-HKU1, HCoV-229E, HAdV and HPeV (Table 4).

## 4. Discussion

In this study, we analyzed the transmission dynamics of respiratory viruses in Mie Prefecture by comparing the number of detections, positivity rates of each virus and outbreak patterns of SARS-CoV-2 variants. Throughout the study period, the prevalence of respiratory viral infections, defined as the detection of at least one respiratory virus, ranged from 37.0% to 86.8% (Figure 2a). Most of the detected viruses followed a pattern of one to two epidemics per year. Immediately after the onset of the COVID-19 pandemic in 2020, there was a notable suppression of respiratory virus circulation, including influenza virus and RSV [22]. However, from 2021 onwards, there was a gradual increase in the detection of other respiratory viruses. Regarding RSV, while pre-pandemic outbreaks typically occurred from late summer to autumn, post-pandemic outbreaks shifted to spring and summer periods, a globally reported phenomenon [23]. This seasonal shift was also confirmed in our study (Figure 2g).

In the present study, 70% of eligible patients were under 5 years of age, and analysis by age revealed a high incidence of respiratory viral infections among infants. In particular, when comparing the group aged <2 years with the other age groups, the proportion of virus non-detection was significantly lower in the <2 age group, and the detection of HRV/EV, RSV, HAdV, HPeV and HBoV was significantly higher (Table 2). These viruses are known to commonly infect children under 5 years old. For RSV in particular, significantly higher rates of severe disease have been reported in infants <6 months of age, emphasizing the need for careful clinical follow-up in this age group [4]. These results also suggest that the relatively weak innate immune responses in infants may contribute to their increased susceptibility to respiratory viruses such as HRV/EV, RSV, HAdV, HPeV and HBoV, particularly when combined with increased exposure opportunities in childcare facilities; however, this immune response pattern appears to differ for SARS-CoV-2 infections, where children exhibit robust innate immune responses in their upper respiratory tract comparable to adults [24,25]. In addition, the behavior patterns specific to infants (e.g., putting things in their mouths, inadequate hand washing) may also increase the risk of viral infection. These findings highlight the necessity of implementing age-appropriate infection control measures.

Regarding the multiplicity of virus detections, single-virus infections were predominant across all age groups under 15 years (“no virus detection” was the most common outcome in those aged ≥15 years) (Table 3). In individuals aged 5 years and older, up to three different viruses were detected simultaneously. Conversely, in the group aged <5 years, 21 out of 841 cases (2.5%) exhibited co-infection with four or more viral species (Table 3). The increased opportunities for simultaneous exposure in community environments contribute to multiple viral detections in infants, though the frequency decreases with increasing viral numbers (Table 3). This inverse relationship reflects the dynamic interplay between exposure opportunities and immune responses: initial viral infection triggers interferon production [26,27], potentially providing partial protection against subsequent infections. This immune modulation is particularly relevant in young children, where innate immunity serves as the primary defense mechanism [28,29,30].

During the specimen collection period, the impact of SARS-CoV-2 on other respiratory viruses appeared to be heterogeneous. Our findings align with multiple studies examining viral circulation patterns during and after the COVID-19 pandemic in different geographical regions [11,31]. Our analysis revealed virus-specific patterns: when SARS-CoV-2 increased (2023, Jan, Feb, March), HRV/EV and RSV showed reduced detection rates, while other viruses (FluA, HPIV3 and HCoVs) maintained their circulation (Figure 2). This observation is consistent with recent studies demonstrating differential effects of SARS-CoV-2 prevalence in various respiratory pathogens [7]. This selective inhibition was supported by co-detection analysis, which showed a notably higher single-detection rate for SARS-CoV-2 (69.2%) compared to HRV/EV and RSV (approximately 50%). These patterns likely reflect complex virus–virus interactions at the cellular level, including the following:

(1) Heterogeneous cellular responses, where infected cells may become more susceptible to secondary infections while uninfected bystander cells develop enhanced resistance through interferon-mediated signaling [32,33].
(2) Differences in cellular tropism and receptor usage among respiratory viruses [34].
(3) Virus-specific transmission characteristics and host immune responses [22].

These findings suggest that the impact of SARS-CoV-2 on respiratory virus circulation is complex and virus-specific, highlighting the importance of continued surveillance study to understand these dynamic interactions.

Among all detected viruses, only HBoV showed a significantly high tendency for co-detection (OR:1.98, 95% CI:1.20–3.26, Table 4). This high co-detection rate (83.6%) in children under 5 years requires careful interpretation, as it may reflect either clinically relevant co-infections affecting disease severity [35,36] or the detection of persistent viral DNA from previous infections [37], a unique characteristic of HBoV. Further investigation is needed to determine the clinical significance of these co-detection patterns.

Considering the association between the simultaneous detection of multiple viruses and severe illness, while some studies have reported a correlation between co-infection and severe illness [38,39], others have found no such association [40]. This discrepancy in findings underscores the complexity of multiple virus infections and their clinical outcomes. Factors such as virus–virus interactions, individual variations in host immune responses, and environmental influences likely play significant roles. To better understand this complexity, future studies should focus on detailed analyses of the pathogenicity of individual viruses and the host immune response. Such investigations could contribute to the development of predictive models for assessing the risk of severe outcomes in cases of multiple virus infection. Additionally, there is a critical need for ongoing research to address the complex challenge of optimizing treatment strategies for patients with multiple concurrent viral infections. For example, reports of co-infections with RSV and HMPV suggest that specific viral combinations may necessitate careful prognostic observations [38].

In this study, our findings demonstrate a statistically significant higher prevalence of cough and runny nose among RSV-positive patients, whereas patients with HRV and/or EV detection exhibited a significantly higher incidence of wheezing and runny nose (*p* < 0.05). Combining clinical symptom data with virus detection information can enable more appropriate medical interventions and a reduction in disease severity.

## 5. Conclusions

In conclusion, the introduction and continuous refinement of this novel surveillance system provides a robust framework for accurately monitoring the dynamics and severity risk of respiratory virus epidemics. These insights are expected to contribute significantly to the development of effective preventive measures and therapeutic strategies, ultimately advancing public health outcomes.

## Figures and Tables

**Table 1:** Summary of specimen type, sex, age, clinical symptoms and physician diagnosis.

	Value
**Specimens**	
Nasopharyngeal swab	1504 (95.6%)
Nasal discharge	69 (4.4%)
**Sex, Male**	899 (57.2%)
**Age**	
Median age, years [IQR]	2.4 [1.1–5.8]
**Age range, years**	
0 to <2	651 (41.4%)
2 to <5	469 (29.8%)
5 to <15	308 (19.6%)
≥15	145 (9.2%)
**Fever**	
No records	650 (41.3%)
≥38 °C	774/923 (83.9%)
**Clinical symptoms** **^1^** **(*n* = 1175)**	
Cough	396 (33.7%)
Runny nose	284 (24.2%)
Sore throat	201 (17.1%)
Wheezing	45 (3.8%)
Fatigue	8 (0.7%)
Not mentioned	11 (0.9%)
**Physician diagnosis**	
Bronchitis and bronchiolitis *	241 (20.5%)
Upper respiratory inflammation	122 (10.4%)
Asthma **	58 (4.9%)
Pneumonia ***	41 (3.5%)

^1^ Excludes 398 specimens collected between April 2021 and March 2022 (see Figure 1); * not including bronchopneumonia; ** not including coughing asthma and asthma-like bronchitis; *** including bronchial pneumonia.

**Table 2:** Number, percentage and median patient age for samples that tested positive for respiratory viruses, as detected by age group between April 2022 and March 2023.

	<2 (*n* = 478), *n* (%)		2 to <5 (*n* = 363), *n* (%)		5 to <15 (*n* = 225), *n* (%)		≥15 (*n* = 109), *n* (%)		Total (n)	Median [IQR]
SARS-CoV-2	65	13.6% **	32	8.8%	42	18.1%	17	15.6%	156	2.7 [0.8–8.9]
		41.7% ***		20.5%		26.9%		10.9%		
FluA	2	0.4%	5	1.4%	8	3.4%	3	2.8%	18	5.8 [3.3–9.8]
		11.1%		27.8%		44.4%		16.7%		
HRV/EV *	154	32.2%	136	37.4%	43	18.5%	9	8.3%	342	2.2 [1.1–3.8]
		45.0%		39.8%		12.6%		2.6%		
HPIV1	10	2.1%	23	6.3%	6	2.6%	0	0.0%	39	3.1 [1.8–4.6]
		25.6%		59.0%		15.4%		0.0%		
HPIV3	50	10.4%	47	12.9%	14	6.0%	0	0.0%	111	2.1 [1.3–3.6]
		45.0%		42.3%		12.6%		0.0%		
HPIV4	2	0.4%	7	1.9%	1	0.4%	0	0.0%	10	4.0 [2.6–4.1]
		20.0%		70.0%		10.0%		0.0%		
HCoV-NL63	9	1.9%	5	1.4%	1	0.4%	2	1.8%	17	1.8 [0.8–4.5]
		52.9%		29.4%		5.9%		11.8%		
HCoV-HKU1	5	1.0%	6	1.6%	1	0.4%	1	0.9%	13	2.1 [1.6–3.6]
		38.5%		46.2%		7.7%		7.7%		
HCoV-229E	0	0.0%	1	0.3%	0	0.0%	1	0.9%	2	25.2 [14.7–35.7]
		0.0%		50.0%		0.0%		50.0%		
HCoV-OC43	15	3.1%	27	7.4%	2	0.9%	8	7.3%	52	2.8 [1.8–4.1]
		28.8%		51.9%		3.8%		15.4%		
RSV	84	17.5%	69	19.0%	7	3.0%	5	4.6%	165	1.9 [1–2.8]
		50.9%		41.8%		4.2%		3.0%		
HAdV	39	8.1%	26	7.1%	7	3.0%	1	0.9%	73	1.7 [1.1–2.8]
		53.4%		35.6%		9.6%		1.4%		
HPMV	22	4.6%	38	10.4%	18	7.8%	5	4.6%	83	3.3 [1.9–5.1]
		26.5%		45.8%		21.7%		6.0%		
HPeV	22	4.6%	11	3.0%	0	0.0%	0	0.0%	33	1.5 [1.1–2.1]
		66.7%		33.3%		0.0%		0.0%		
HBoV	69	14.4%	51	14.0%	2	0.9%	0	0.0%	122	1.8 [1.3–2.6]
		56.6%		41.8%		1.6%		0.0%		

Abbreviations: FluA, influenza virus type A; HRV, human rhinovirus; EV, enterovirus; HPIV, human parainfluenza virus; HCoV, human coronavirus; RSV, respiratory syncytial virus; HAdV, human adenovirus; HMPV, human metapneumovirus A/B; HPeV, human parechovirus; HBoV, human bocavirus. * Either HRV or EV or both were detected. ** Percentage within each age group. *** Percentage among the total detections.

**Table 3:** Number and percentage of co-detected viruses by age group between April 2022 and March 2023.

Number of Viruses Detected	<2 (*n* = 478)		2 to <5 (*n* = 363)		5 to <15 (*n* = 225)		≥15 (*n* = 109)	
	N	(%)	N	(%)	N	(%)	N	(%)
0	92	19.3	47	12.9	94	41.8	60	55.1
1	263	55.0	194	53.4	114	50.7	46	42.2
2	95	19.9	90	24.8	13	5.8	3	2.8
3	18	3.8	21	5.8	4	1.8	0	0
4	9	1.9	9	2.5	0	0	0	0
5	1	0.2	1	0.3	0	0	0	0
6	0	0	1	0.3	0	0	0	0

**Table 4:** Number, percentage and odds ratio of single or multiple respiratory viruses detected between April 2022 and March 2023.

	Number and Percentage of Single or Multiple Detections				Total	Odds Ratio	95% Confidence Interval
	Single Detection	(%)	Detection with Other Virus(es)	(%)			
SARS-CoV-2	108	69.2	48	30.8	156	0.18	0.13–0.26
FluA	14	77.8	4	22.2	18	0.10	0.03–0.30
HRV/EV *	185	54.1	157	45.9	342	0.46	0.36–0.60
HPIV1	25	64.1	14	35.9	39	0.20	0.10–0.38
HPIV3	53	47.7	58	52.3	111	0.42	0.28–0.62
HPIV4	1	10.0	9	90.0	10	3.04	0.38–24.0
HCoV-NL63	9	52.9	8	47.1	17	0.30	0.11–0.79
HCoV-HKU1	6	46.2	7	53.8	13	0.40	0.13–1.19
HCoV-229E	1	50.0	1	50.0	2	0.33	0.02–5.38
HCoV-OC43	28	53.8	24	46.2	52	0.30	0.17–0.53
RSV	95	57.6	70	42.4	165	0.30	0.22–0.42
HAdV	23	31.5	50	68.5	73	0.79	0.48–1.32
HMPV	41	49.4	42	50.6	83	0.38	0.24–0.59
HPeV	8	24.2	25	75.8	33	1.09	0.48–2.43
HBoV	20	16.4	102	83.6	122	1.98	1.20–3.26

Abbreviations: FluA, influenza virus type A; HRV, human rhinovirus; EV, enterovirus; HPIV, human parainfluenza virus; HCoV, human coronavirus; RSV, respiratory syncytial virus; HAdV, human adenovirus; HMPV, human metapneumovirus A/B; HPeV, human parechovirus; HBoV, human bocavirus. * Either HRV or EV or both were detected.

## Data Availability

The data are not publicly available due to privacy or ethical restrictions.
